# Health-related quality of life assessment in Indonesian childhood acute lymphoblastic leukemia

**DOI:** 10.1186/1477-7525-6-96

**Published:** 2008-11-09

**Authors:** Mei N Sitaresmi, Saskia Mostert, Chad M Gundy, Sutaryo, Anjo JP Veerman

**Affiliations:** 1 Department of Pediatrics, Faculty of Medicine, Gadjah Mada University, Yogyakarta, Indonesia; 2 Department of Pediatric Hematology-Oncology, VU Medical Center, VU, Amsterdam, the Netherlands; 3 Department of Psycho-Social Research and Epidemiology, Dutch Cancer Institute-Antoni van Leeuwenhoek Hospital, Amsterdam, the Netherlands; 4 Department of Pediatric Hematology-Oncology, Faculty of Medicine, Gadjah Mada University, Yogyakarta, Indonesia

## Abstract

**Background:**

Most studies on Health-related Quality of Life (HRQOL) in children with cancer were conducted in developed countries. The aims of this study were to assess the HRQOL in childhood acute lymphoblastic leukemia (ALL) patients in Indonesia and to assess the influence of demographic and medical characteristics on HRQOL.

**Methods:**

After cultural linguistic validation, a cross-sectional study of HRQOL was conducted with childhood ALL patients and their guardians in various phases of treatment using the Pediatric Quality of Life Inventory™ (PedsQL™) 4.0 Generic Core Scale and the Pediatric Quality of Life Inventory™ (PedsQL™) 3.0 Cancer Module.

**Results:**

Ninety-eight guardians and 55 patients participated. The internal consistency of both scales ranged from 0.57 to 0.92. HRQOL of Indonesian patients was comparable with those in developed countries. There were moderate to good correlations between self-reports and proxy-reports, however guardians tended to report worse HRQOL than patients. Children of the 2–5 year-group significantly had more problems in procedural anxiety, treatment anxiety and communication subscales than in older groups (p < 0.05). In the non-intensive phase HRQOL was significantly better than in the intensive phase, both in patient self-reports and proxy-reports.

**Conclusion:**

Younger children had more problems in procedural anxiety, treatment anxiety and communication subscales. Therefore, special care during intervention procedures is needed to promote their normal development. Psychosocial support should be provided to children and their parents to facilitate their coping with disease and its treatment.

## Background

The survival rate of acute lymphoblastic leukaemia (ALL), the most common malignancy in children, has improved in recent years to a 5-year survival rate of about 80% [1,2], although it is often less than 35% in developing countries [3-6]. As the treatment for childhood ALL can be aggressive and associated with acute and long-term morbidity due to side effects, it is important to not only look at the survival but to analyze the health-related quality of life (HRQOL) as well [2]. Implementing HRQOL measurements in pediatric healthcare settings can improve communication between patients and health care providers (HCP), increase patient/parent satisfaction with HCP, identify hidden morbidities, and assist in clinical decision-making [7,8].

HRQOL is defined as a multidimensional construct composed of the patients' perceptions of the impact of disease and treatment on his or her functioning in a variety of aspects of life, including physical, psychological and social health domains [8,9]. Pediatric patient self-reports are considered to be the standard for measuring HRQOL, however proxy reports may be the only available source of data when children are too young, too cognitively impaired or too sick to complete a HRQOL instrument [10,11]. A generic HRQOL measurement instrument can be used in a healthy population and enables standardized comparisons between healthy children and children with chronic health problems. However it cannot be used to measure specific disease symptoms and treatment side effects. A disease-specific instrument is unable to provide comparisons with healthy children, but it is more sensitive to measure clinical symptoms of diseases. Using both a generic and specific diseases instrument is recommended to achieve a more comprehensive evaluation of the patients' HRQOL [8]. The Pediatric Quality of Life Inventory (PedsQL™) 4.0 Generic Core Scales [12], and the Pediatric Quality of Life Inventory™ (PedsQL) 3.0 Cancer Module [13] are considered to be very promising HRQOL instruments for children and adolescents, integrating generic core scales and disease-specific modules into one measurement system. It is designed for pediatric patients between the ages of 2 and 18 years, and available in a patient self-report version designed for children/adolescents and a proxy-report version for guardians [14-17].

Some studies found that there was correlation between gender and socioeconomic status (SES) and health status influence HRQOL [14,17]. Many studies assessed HRQOL in childhood ALL patients in developed countries, but few have been conducted in developing countries, where supportive care for ALL patients is limited [18-21]. The aims of this study were to assess the HRQOL in childhood acute lymphoblastic leukemia (ALL) patients in Indonesia and to assess the influence of demographic and medical characteristics on HRQOL.

## Methods

### Setting

Indonesia is a multi-island state, and has a population of approximately 220 million of whom 37% are children under 15 years. A childhood leukemia incidence of 2.5 to 4.0 new cases per 100 000 children leads to an estimated 2000 to 3200 new childhood ALL cases each year [22]. Our study was conducted at the pediatric department of Dr. Sardjito Hospital in Yogyakarta, Indonesia. Dr. Sardjito Hospital is a teaching hospital of the medical faculty of the Gadjah Mada University, and a tertiary-care referral hospital of the Yogyakarta and Central Java Provinces. Annually aproximately 30–40 children are diagnosed with ALL. The pediatric department consists of a clinic (VIP, 1^st^, 2^nd^, and 3^rd ^class) and a policlinic (VIP and general).

### Study design

After cultural linguistic validation, a cross-sectional study was conducted with childhood ALL patients and their guardians in various phases of treatment using the Pediatric Quality of Life Inventory™ (PedsQL™) 4.0 Generic Core Scale and the Pediatric Quality of Life Inventory™ (PedsQL™) 3.0 Cancer Module. The cultural linguistic validation process consists of 3 steps: forward translation, backward translation, and patient testing. In the first step, the instruments were translated from English version to Bahasa Indonesia version, and it was done independently by two local professional translators, native Indonesia speakers, and bilingual in English language. The second step, the Bahasa Indonesia version was translated back to English version and it was done independently by two local professional translators, native English speakers, and bilingual in Bahasa Indonesia language. In this step a comparison between the backward version with the original version was assessed in order to detect any misunderstandings, mistranslations or inaccuracies in the intermediary forward version of the questionnaire. Finally, the translated questionnaire was tested in 5 cancer families, both in children and their guardians, to determine whether the translation (instructions, items and response choices) is acceptable, whether it is understood in the way it is supposed to be, and whether the language used is simple and appropriate. All PedsQL™ translations were conducted in close ongoing collaboration with Mapi Research Institute in Lyon, France and with Dr. James W. Varni.

Participants were childhood ALL patients who were hospitalized or visited the policlinic at Dr Sardjito Hospital, Yogyakarta, Indonesia, from October 2006 until January 2008. Eligible patients were children of 2–16 years at time of diagnosis. Patients with severe conditions who were not able to answer the questionnaire were excluded. During this period 2 ALL dexametasone-based protocols were used: Wijaya Kusuma ALL protocol and Indonesia ALL protocol. According to both protocols there are 2 risk groups: standard risk (SR) and high risk (HR). Both protocols consist of an induction, consolidation, re-induction (only for HR) and maintenance phase. We classified the phase as either intensive (induction, consolidation, re-induction) or non-intensive (maintenance). Interviews of eligible patients and their guardians, who were interviewed separately, were performed individually by a trained psychologist. It took 10–20 minutes for guardians and 20–45 minutes for patients to complete the questionnaire. Demographic data regarding information about age, relationship of respondent to child, number of children in the family, parental educational status, and socio-economic status were collected from guardians. Parental educational status was categorized into low educational status (no education, elementary school, junior high school) and high educational status (senior high school, academy, university). We classified SES as either poor or prosperous. This classification was based on 2 determinants: monthly income level of parents and assigned hospital class during the diagnostic process. Both determinants are obtained routinely during admission to the clinic and recorded in the medical records. The threshold per month income for poor versus prosperous families was set at 1.000.000 Indonesian Rupiah (about 100 US dollar). Patients attending VIP or first class wards and VIP policlinic were classified as prosperous. In case there is discordance between family income and hospitalization class or data of family income is not available, we used hospitalization class as SES determinant. Medical data regarding diagnosis and treatment status were obtained from the patients' MR. The study was approved by the Medical Ethics Committees of Faculty medicine, Gadjah Mada University. Informed consents were also obtained from the guardians.

### Instruments and measurements

The PedsQL™ 4.0 Generic Core Scale (Generic Scale) is a multidimensional instrument developed by Varni et al [12]. It consists of 23 items categorized into 4 subscales: physical functioning (8 items), emotional functioning (5 items), social functioning (5 items) and school functioning (5 items). For interpretation, three scores can be obtained: physical health (score of the physical functioning subscale); psychosocial health (combined scores of the emotional functioning, social functioning and school functioning subscales), and total score (combined score of physical health and psychosocial health).

The PedsQL™ 3.0 Cancer Module (Cancer Module) is a multidimensional instrument developed by Varni et al. to assess the impact of disease and treatment on the HRQOL of pediatric cancer patients [13]. It consists of 27 items distributed to 8 subscales: pain and hurt (2 items), nausea (5 items), procedural anxiety (3 items), treatment anxiety (3 items), worry (3 items), cognitive problems (5 items), perceived physical appearance (3 items) and communication (3 items). Both instruments were available for children aged: 5–7, 8–12 and 13–18 years old; as well as for the guardians of children aged: 2–4, 5–7, 8–12 and 13–18 years old.

The scale has five Likert response options: never, almost never, sometimes, often and almost always. To simplify the interpretation, all Likert scales were converted to 0–100. The higher scores indicate a higher HRQOL. For the versions adapted to children between the ages of 5 and 7 years, there are only three response options: never, sometimes and almost always (corresponding to 100, 50, 0). For this age, a Face Scale was used, comprising of 3 pictures of facial expressions varying from a smiling face to a very sad face to indicate no problem/difficulty/pain to a lot of problems/difficulty/pain.

### Statistical analysis

The internal consistency of the Generic Scales and the Cancer Scale were measured by Cronbach's Alpha Coefficient. Values ≥ 0.70 were considered acceptable for comparisons between groups [23]. The correlation between the self-reports and the proxy-reports was assessed by interclass correlation coefficient. A comparison score between different demographic characteristics: age, gender, parental education status, SES, position of the child in the family, and medical groups of treatment (risk stratification, phase of treatment) were measured using Independent-Sample T test. All analyses were performed with two-sided tests and a value of p < 0.05 was considered significant. The SPSS for Windows (version 12) programs were used for the data analysis.

## Results

### Demographic and medical characteristics

During October 2006 – January 2008, 110 ALL patients were hospitalized or visited the pediatric department of Dr. Sardjito Hospital. Ninety-eight patients were recruited according to the inclusion and exclusion criteria. There were no significant differences between the studied group and those who did not participate with regard to demographic characteristics. All guardians completed the proxy-reports questionnaire. Fifty-five patients participated in patient self-report, 3 (10%) patients of 5–7 year-old age group did not wish to answer the questionnaire. There was no significant difference between proxy-reports and self-reports with regard to age, gender, parental educational status, SES, ALL risk stratification, and phase of treatment. The average age of all patients was 6.6 years (SD = 3.7), median 6 years, range 2–16 years. Most respondents of proxy reports were mothers (n = 74, 76%), and most families (n = 76, 78%) belonged to low SES (Table 1).

**Table 1:** Demographic and medical characteristics

Variable	Self-reports (n = 55) n (%)	Proxy-reports (n = 98) n (%)
**Demographic characteristic**		
Child age in year, mean + SD (range)	8.8+3.1(5–16)	6.6 +3.7 (2–16)
2–4 year-group		40 (41)
5–7 year-group	24 (44)	25 (26)
8–12 year-group	24 (44)	25 (26)
13–18 year-group	7 (12)	7 (7)
Child gender, male n (%)	27 (49)	54 (55)
Number of child in family		
single child n (%)	16 (29)	34 (35)
Respondent, n (%)		
mothers		75
father		20
others		4
**Socio-economic status**		
Class of hospitalization: 2^nd ^and 3^rd ^class	42 (76)	76 (78)
Parents' income: <100 US dollar	37 (67)	70 (71)
Parents' employment: non employed	29 (53)	54 (55)
Parents' educational level		
mother: low educational level	27 (49)	74 (76)
father: low educational level	24 (43)	40 (41)
**Medical characteristics**		
Phase of treatment: intensive phase	27 (49)	56 (57)
Risk stratification: SR	24 (44)	49 (50)

### Psychometrics of the questionnaire

Cronbach's alpha for total scale of the Generic Scales and the Cancer Scale were all above 0.7 in both self-reports and proxy-reports. A positive correlation between self-reports and proxy-reports was found on all subscales and total scale of both the Generic Scales and the Cancer Scale, except the perceived physical appearance. Physical functioning, procedural anxiety and communication subscales showed a good correlation between self-reports and proxy-reports (ICC> 0.61), but other subscales only showed a weak-moderate correlation (ICC 0.31–0.60). Guardians tended to report lower HRQOL than did patients in Cancer Scale. In both self report and proxy report, psychosocial health subscale was better than physical health subscale (table 2).

**Table 2:** Scale description and internal consistency reliability for PedsQL generic core scale and cancer module

Scale descriptive	Child self reports (n = 55)			Proxy reports (n = 98)			Patients-proxy scale
	Mean	SD*	α **	Mean	SD*	α **	ICC***
**Generic Scale**							
Total score	71.8	17.8	0.86	71.3	17.2	0.92	0.62
Physical health	68.1	26.4	0.83	64.3	26.6	0.87	0.61
Psychosocial health	73.4	17.3	0.81	73.2	16.0	0.88	0.56
Emotional functioning	71.2	22.1	0.69	69.1	21.8	0.78	0.46
Social functioning	83.4	16.6	0.57	85.0	16.0	0.67	0.36
School Functioning****	63.5	24.6	0.66	61.6	26.0	0.79	0.44
							
**Cancer Scale**							
Total score	77.1	16.8	0.82	72.2	18.3	0.89	0.62
Pain and hurt	80.1	27.4	0.70	71.0	29.8	0.84	0.31
Nausea	82.6	21.4	0.87	78.8	19.5	0.74	0.54
Procedural anxiety	69.9	32.3	0.85	60.1	36.9	0.89	0.61
Treatment anxiety	88.2	22.5	0.78	74.4	33.2	0.90	0.33
Worry	75.5	27.7	0.73	74.6	25.8	0.80	0.33
Cognitive problem	76.9	23,2	0.88	77.6	23.9	0.91	0.60
Perceived physical appear	76.4	27.2	0.64	82.5	22.6	0.69	0.16 NS
communication	68.3	37.0	0.83	60.2	36.9	0.84	0.70

Higher score indicate higher quality of life or fewer problems.

*SD = standard deviation; **α = internal consistency; ***ICC = Interclass correlation coefficient between proxy-reports and child-reports; ****n = 46 in child reports and n = 64 in proxy report.

NS = no significant

### Influence of demographic and medical characteristics on HRQOL

Gender, position of the child in the family, SES, parental educational status, and risk stratification did not correlate to any scales of HRQOL (table 3 and additional file 1). However, HRQOL of 2–5 year-group was significantly lower than HRQOL in the older group regarding procedural anxiety, treatment anxiety and communication subscales (p < 0.05) (table 4). The total score of the Generic Scales and the Cancer Scale in the non-intensive phase were significantly better than in the intensive-phase, in both patient self-reports as well as proxy-reports. Procedural anxiety subscale showed the greatest significant difference between the different phases. In the intensive phase, both children and guardians reported lower scores in procedural anxiety and communication. In the non-intensive phase, both children and guardians reported the lowest score in the worry subscale (table 5).

**Table 3:** Comparison means score of HRQOL (generic scale) between different demographic and medical characteristics: proxy report

	Total Score Mean (SD)	Physical F Mean (SD)	Emotional F Mean (SD)	Social F Mean (SD)	School Mean (SD)
	*P*	*p*	*p*	*p*	*p*
Gender					
Male (n = 54)	72 (16)	66 (27)	70 (21)	87 (14)	63 (23)
Female (n = 44)	69 (19)	62 (26)	67 (22)	83 (18)	60 (29)
*P*	*0.41*	*0.45*	0.61	0.21	0.64
Risk stratification					
SR (n = 49)	72 (17)	66 (26)	69 (28)	84 (17)	66 (24)
HR (n = 49)	69 (17)	62 (26)	68 (22)	86 (15)	57 (27)
*P*	0.45	0.42	0.79	0.42	0.21
SES					
Low (n = 76)	71 (9)	65 (14)	69 (22)	85 (17)	63 (27)
High (n = 22)	69 (11)	64 (18)	70 (20)	85 (16)	56 (22)
*p*	0.78	0.88	0.88	0.90	0.34
Father's occupation					
Unemployed (n = 54)	70 (19)	64 (29)	69 (22)	84 (17)	60 (30)
Employed (n = 44)	71 (15)	62 (26)	68 (21)	86 (15)	63 (21)
*p*	0.84	0.94	0.91	0.42	0.61
Mother's occupation					
Unemployed (n = 74)	71 (18)	66 (28)	68 (21)	85 (17)	62 (23)
Employed (n = 24)	69 (15)	60 (22)	70 (22)	85 (15)	60 (22)
*p*	0.65	0.34	0.69	0.98	0.82
Father's Education					
Low (n = 40)	73 (19)	62 (25)	74 (21)	87 (17)	63 (26)
High (n = 58)	68 (16)	62 (25)	66 (21)	84 (16)	60 (26)
*p*	0.17	0.29	0.09	0.34	0.68
Mother's education					
Low (n = 42)	70 (18)	62 (28)	68 (22)	86 (16)	65 (24)
High (n = 56)	71 (17)	66 (25)	69 (21)	84 (17)	58 (27)
*p*	0.96	0.88	0.74	0.48	0.34

**Table 4:** Comparison means score of HRQOL between difference ages, proxy reports

Descriptive scale	2–4 year n = 40 Mean(SD)	5–16 year n = 58 Mean(SD)	Mean Difference (95% CI)	p
**Generic Scale**				
Total score	70 (18)	71 (17)	1(-6–8)	0.77
Physical functioning	64 (28)	65 (25)	1(-9–12)	0.83
Psychosocial F	73 (16)	73 (16)	0 (-6–7)	0.98
Emotional F	66 (20)	71 (22)	5(-3–14)	0.23
Social F	85 (16)	85 (17)	0 (-6–7)	0.88
School F*	52 (19)	63 (25)	1(-6–29)	0.19
**Cancer Scale**				
Total score	67 (19)	76 (16)	10 (9–17)	0.01
Pain and hurt	70 (32)	72 (27)	2(-9–15)	0.64
Nausea	78 (17)	79 (20)	1 (-6–9)	0.75
Procedural anxiety	44 (36)	71 (33)	27 (12–40)	0.001
Treatment anxiety	57 (39)	86 (21)	29 (16–41)	0.001
Worry	74 (28)	75 (24)	1 (-11-11)	0.88
Cognitive problem	77 (26)	78 (23)	1 (-9–10)	0.72
Perceived physical app.	84 (21)	82 (23)	6 (-10-7)	0.58
communication	49 (28)	68 (34)	19 (5–34)	0.01

Higher score indicate higher quality of life or fewer problems.

*n = 10 in 2–4 year-group and n = 53 in 5–16 year-group

**Table 5:** Comparison means score of HRQOL (generic scale and cancer scale) between intensive and non intensive treatment: self-reports and proxy report (bold)

Descriptive scale	Intensive* Mean(SD)	Non intensive* Mean(SD)	Mean Difference (95% CI)	*p*
**Generic Scale**				
Total score	63(17)	79(14)	16 (7–25)	0.01
	**61 (14)**	**83 (17)**	**22 (16–28)**	**0.001**
Physical Health	57(26)	77(22)	20 (–-34)	0.001
	**50 (23)**	**83 (17)**	**33 (24–41)**	**0.001**
Psychosocial Health	65 (17)	80 (13)	15 (6–23)	0.01
	**66 (14)**	**83 (12)**	**17 (12–23)**	**0.01**
Emotional Functioning	64 (25)	76 (18)	12 (-1–23)	0.06
	**61 (21)**	**80 (16)**	**21 (12–28)**	**0.001**
Social Functioning	78 (18)	88 (13)	10 (2–19)	0.01
	**79 (17)**	**92 (13)**	**13 (7–39)**	**0.01**
School Functioning**	50 (25)	75(17)	25 (13–19)	0.001
	**47 (26)**	**75 (16)**	**28 (17–39)**	**0.001**
**Cancer Scale**				
Total score	69 (13)	84 (16)	15 (7–24)	0.01
	**63 (15)**	**85 (12)**	**22 (17–28)**	**0.001**
Pain and hurt	72 (26)	88 (26)	16 (2–31)	0.01
	**59 (31)**	**87 (17)**	**38 (18–39)**	**0.001**
Nausea	81 (20)	84 (22)	3 (-8–14)	0.63
	**76 (20)**	**83 (17)**	**7 (-1–14)**	**0.07**
Procedural anxiety	54 (29)	85 (27)	31 (12–47)	0.001
	**43 (34)**	**82 (27)**	**39 (26–53)**	**0.001**
Treatment anxiety	84 (23)	92 (21)	8 (-4–20)	0.19
	**64 (36)**	**89 (16)**	**25 (13–38)**	**0.001**
Worry	74 (26)	76 (29)	2 (-13–18)	0.75
	**68 (23)**	**81 (23)**	**13 (2–23)**	**0.01**
Cognitive problem	71 (23)	82 (21)	11 (-1–23)	0.07
	**73 (24)**	**82 (22)**	**9 (1–20)**	**0.04**
Perceived physical app.	71 (28)	81 (25)	9 (-5–24)	0.21
	**75 (23)**	**93 (16)**	**18 (9–26)**	**0.001**
Communication	48 (26)	87 (26)	39 (22–57)	0.001
	**43 (34)**	**82 (21)**	**39 (26–52)**	**0.001**

Higher score indicate higher quality of life or fewer problems; *self-reports (n = 27 in the intensive phase and n = 28 in the non-intensive phase); proxy-reports (n = 56 in the intensive phase and n = 42 in the non-intensive phase), ** self-reports (n = 22 in intensive treatment and n = 24 in non intensive treatment); proxy-reports (n = 31 in intensive treatment and n = 33 in non intensive treatment)

## Discussion

Although recent survival rates of childhood acute lymphoblastic leukemia (ALL) in developed countries are about 80%, the survival rates in developing countries are often less than 35%. In our previous study the overall even-free survival rate was only 20% [24]. One might assume that the low survival in developing countries may result in lower HRQOL due to increased morbidity and poor socio-economic circumstances. It is surprising therefore, that the scores of the Generic Scale in our study were in fact similar than reported in studies conducted in developed countries using the same instrument [19,25,26]. A reason for this might be that our children and their guardians expect a lower HRQOL as a normal consequence of having a severe disease.

There was no significant difference on HRQOL between different SES and parent educational status. Other study found the similar results [19]. However, we did not find any difference on HRQOL between boys and girls while other studies found that girls had more problems in the emotional domain [19,20]. Our study showed that younger children had lower HRQOL in procedural anxiety, treatment anxiety and communication subscales. Therefore, these younger children need special care, especially in the area of interventions: vena-punctures, bone marrow puncture and lumbar puncture. Dynamic psychological and social development of 2–5 year-old children can be interrupted by serious life-threatening illness, regular invasive examination and hospital visits, interruption of school attendance and social activities, as well as family crisis. All these factors can influence and interrupt normal psychological, social development and further academic achievement [16].

In both self-reports and proxy-reports, the psychosocial health subscale was better than the physical health subscale. This result was similar to other studies assessing HRQOL in ALL patients on treatment [19,25]. In contrast, studies on survivors of ALL found that psychosocial health was lower than physical health [27,28]. Although survivors of ALL were generally well, they experienced considerable impact on psychosocial health. Survivors may be worried about possible late-effects and recurrence of cancer. This finding implies that psychosocial support remains important long after treatment has completed, and even when the physical health appears well.

As can be expected, HRQOL scores in the non-intensive phase were generally significantly better than in the intensive-phase, in both self-reports and proxy-reports. Significant differences were found in the physical health, pain, procedural anxiety and communication subscales. These results are similar to earlier studies [10,20,25]. In the intensive phase, children had more problems on physical functioning and pain subscales than in the non-intensive phase. It may be due to the treatment, where more aggressive chemotherapy was given in the intensive phase or due to the disease itself. Most of our patients come to the hospital in an advanced stage of the disease after having been hospitalized and treated by previous hospitals. Better scores, less problems, in the non-intensive phase on procedural anxiety and communication may be due to the fact that the patients had already adapted with procedures and HCP.

In the non-intensive phase, both patients and their guardians perceived that children had lower scores, more problems, in worry and emotional subscale. It is not surprising that patients still worry about side-effects of the chemotherapy and curability of ALL since many patients in our hospital suffer from severe side-effects or die during the course of treatment. This concern about side-effects and curability may be a reason for non-compliance. Therefore it is important that health care providers improve their quality of care to reduce side-effects and improve survival rate.

We, like others [10,17,27]. found that there were moderate to good correlations between self-reports and proxy reports. Parents tended to perceive their child's HRQOL worse than children themselves. This may reflect parents-distress regarding the condition of their children. More emphasis on psychological support for both patients and parents is needed.

In summary, younger children had more problems in procedural anxiety, treatment anxiety and communication subscales. Therefore, special care during intervention procedures is needed to promote their normal development. Psychosocial support should be provided for both children and their guardians to facilitate their coping with the disease and its treatment.

## Abbreviations

ALL: acute lymphoblastic leukemia; HRQOL: health-related quality of life; PedsQL™: the Pediatric Quality of Life Inventory™.

## Competing interests

The authors declare that they have no competing interests.

## Authors' contributions

All authors collaborated in the study design; MNS collected the data; MNS and CG conducted the analysis; MNS, SM, CG, ST, AV drafted the paper; and all authors reviewed and approved the manuscript.

## Supplementary Material

Additional file 1 **Comparison means score of HRQOL (cancer module) between different demographic and medical characteristics: proxy report.** Click here for file
