# Supplementary material for: Health-related quality of life assessment in Indonesian childhood acute lymphoblastic leukemia
Source: Health Qual Life Outcomes. 2008 Nov 9;6:96. doi: 10.1186/1477-7525-6-96 (PMC2613134; doi:10.1186/1477-7525-6-96)
Supplement: Additional file 1 — Comparison means score of HRQOL (cancer module) between different demographic and medical characteristics: proxy report. [file 1477-7525-6-96-S1.doc]

Table 4 Comparison means score of HRQOL (cancer module) between different demographic and medical characteristics: proxy report

|  | Total  M (SD)  *P* | Pain  M (SD)  *P* | Nausea  M (SD)  *P* | Procedural  M (SD)  *P* | Treatment  M (SD)  *P* | Worry  M (SD)  *P* | Cognitive  M (SD)  *P* | Perceive  Physical app  M (SD)  *P* | Commu-nication  M (SD)  *P* |
| --- | --- | --- | --- | --- | --- | --- | --- | --- | --- |
| Gender  Male (n=54)  Female (n=44)  *P*  Risk stratification  SR (n=49)  HR (n=49)  *p*  SES  Low (n=76)  High (n=22)  *p*  Father’s occupation  Unemployed (n=54)  employed (n=44)  *p*  Mother’s occupation  Unemployed (n=74)  Employed (n=24)  *p*  Father’s Education  Low (n=40)  High (n=58)  *p*  Mother’s education  Low (n=42)  High (n=56)  *p* | 71 (19)  73 (16)  0.57  73 (18)  71 (18)  0.58  73 (19)  71 (15)  0.72  75 (17)  68 (18)  0.08  72 (18)  73 (17)  0.81  76 (16)  69 (19)  0.06  71 (16)  72 (19)  0.87 | 73 (25)  70 (34)  0.69  72 (30)  70 (29)  0.83  71 (30)  71 (29)  0.99  74 (28)  68 (30)  0.31  72 (29)  69 (32)  0.69  74 (27)  69 (31)  0.41  70 (29)  72 (30)  0.82 | 79 (19)  78 (20)  0.82  79 (20)  79 (18)  0.99  80 (18)  73 (21)  0.14  80 (18)  77 (20)  0.53  80 (19)  76 (19)  0.48  81 (17)  77 (20)  0.28  79 (20)  78 (19)  0.72 | 60 (39)  59 (34)  0.96  62 (26)  57 (27)  0.57  62 (38)  54 (32)  0.39  70 (34)  52 (37)  0.04  60 (37)  59 (34)  0.94  69 (34)  54 (37)  0.06  59 (37)  61 (36)  0.78 | 71 (36)  78 (18)  0.26  76 (31)  72 (35)  0.54  74 (34)  75 (30)  0.84  77 (34)  70 (34)  0.28  73 (34)  79 (29)  0.37  77 (32)  70 (34)  0.28  73 (32)  70 (34)  0.28 | 74 (25)  75 (26)  0.95  76 (31)  76 (25)  0.63  75 (26)  74 (22)  0.93  76 (24)  71 (26)  0.24  76 (25)  69 (25)  0.28  77 (25)  72 (26)  0.38  77 (24)  73 (26)  0.55 | 77 (24)  78 (23)  0.88  76 (25)  80 (21)  0.39  77 (24)  78 (23)  0.88  79 (23)  77 (24)  0.70  76 (24)  82 (20)  0.31  78 (23)  77 (24)  0.96  76 (23)  79 (24)  0.59 | 82 (21)  83 (24)  0.77  85 (21)  80 (23)  0.39  82 (23)  83 (21)  0.86  87 (14)  79 (24)  0.12  81 (23)  87 (20)  0.29  84 (21)  81 (23)  0.51  83 (22)  82 (23)  0.77 | 57 (38)  65 (35)  0.28  64 (35)  56 (37)  0.27  60 (37)  59 (34)  0.97  64 (37)  56 (36)  0.29  59 (37)  62 (34)  0.72  63  58  0.46  57  62  0.53 |

M (SD)= mean (standard deviation); *p*= level of significant
